# Plant-parasitic nematodes respond to root exudate signals with host-specific gene expression patterns

**DOI:** 10.1371/journal.ppat.1007503

**Published:** 2019-02-01

**Authors:** Christopher A. Bell, Catherine J. Lilley, James McCarthy, Howard J. Atkinson, P. E. Urwin

**Affiliations:** 1 Faculty of Biological Sciences, University of Leeds, Leeds, United Kingdom; 2 Nestle R.D., Tours, France; University of Wisconsin - Madison, UNITED STATES

## Abstract

Plant parasitic nematodes must be able to locate and feed from their host in order to survive. Here we show that *Pratylenchus coffeae* regulates the expression of selected cell-wall degrading enzyme genes relative to the abundance of substrate in root exudates, thereby tailoring gene expression for root entry of the immediate host. The concentration of cellulose or xylan within the exudate determined the level of β-1,4-endoglucanase (*Pc-eng-1*) and β-1,4-endoxylanase (*Pc-xyl*) upregulation respectively. Treatment of *P*. *coffeae* with cellulose or xylan or with root exudates deficient in cellulose or xylan conferred a specific gene expression response of *Pc-eng-1* or *Pc-xyl* respectively with no effect on expression of another cell wall degrading enzyme gene, a pectate lyase (*Pc-pel)*. RNA interference confirmed the importance of regulating these genes as lowered transcript levels reduced root penetration by the nematode. Gene expression in this plant parasitic nematode is therefore influenced, in a host-specific manner, by cell wall components that are either secreted by the plant or released by degradation of root tissue. Transcriptional plasticity may have evolved as an adaptation for host recognition and increased root invasion by this polyphagous species.

## Author summary

Plant parasitic nematodes feed from plant roots to support their development. In order to enter and reproduce within the host the nematode must interact with the components exuded by the root. The components are known to vary between individual plant species thus presenting different challenges for the nematode. We observe upregulation of two cell wall degrading enzyme genes in *Pratylenchus coffeae* upon exposure to root exudates. The nematode genes are differentially expressed dependent upon the identity of the plant. The relative expression of each gene correlates with the abundance of the encoded enzyme substrate in the nematode environment, indicating that the nematode perceives these components and responds by tailoring gene expression for what is currently required for host-parasitism. This ability may explain the wide host range of this nematode species and may be shared by other parasites.

## Introduction

Plant pathogens must recognise and respond to host signals in order to survive, with root exudates particularly important for those that are soil-borne [1,2,3]. Root exudates contain up to 20% of the plant’s photosynthetically fixed carbon in the form of sugars, amino acids, organic acids, proteins and carbohydrates with the composition varying between plant species [4,5]. Plant parasitic nematodes are amongst the four most economically important groups of plant pathogens, causing >$80 billion of damage to crops globally each year [6]. They are principally root parasites and root exudates play an important role in host-nematode interactions, inducing nematode hatching and thrusting of an anterior, hollow stylet used to penetrate plant cell walls and during feeding [7, 8, 9]. Nematodes orientate to plant roots in response to chemical gradients (e.g. monosaccharides, carbon dioxide, volatile organic compounds and amino acids) provided by root exudates [7]. Plant hormones such as ethylene and auxin, and their signalling pathways, have been implicated in the attractiveness of roots towards nematodes [10, 11, 12].

This group of pathogens contains species that are host specialists and others that are capable of parasitising many plant species [13]. *Pratylenchus coffeae* is a polyphagous, migratory endoparasitic nematode that uses its stylet to disrupt root tissue mechanically and invade host roots [14]. This process is facilitated by secretion of a range of enzymes, produced in the nematode’s pharyngeal gland cells, which weaken the cell wall [15, 16, 17]. The nematode feeds from a cell by ingesting nutrient rich cytoplasm through its stylet, before entering that cell and proceeding to the next [18]. Intracellular migration of the nematode through the cortical tissue results in root necrosis and nutrient deficiency throughout the host whilst increasing susceptibility to secondary root pathogens [19].

The sedentary plant-parasitic nematode *Meloidogyne incognita* differentially expresses genes in response to *Arabidopsis thaliana* roots and root exudates compared to when not exposed to a host [20]. Also, expression of a β-1,4-endoglucanase in the foliar nematode *Aphelenchoides fragariae* decreases after change of food source from plant to fungal culture [21]. This indicates that nematodes show a degree of transcriptional alteration when presented with a host, however the effect of different host plants and their exudates on nematode responses is unexplored. There is a precedent for some other plant pathogens and pests altering gene expression in response to a particular host. Expression of genes responsible for metabolism, chemotaxis and protein secretion in *Pseudomonas aeruginosa* is significantly altered post-exposure to exudates from different varieties of *Beta vulgaris* [22]. Transcriptional plasticity of multigene clusters also underpins the ability of the peach potato aphid *Myzus persicae* to colonise diverse plant species rapidly [23]. Non-pathogenic symbionts can also respond to particular plant partners. For instance arbuscular mycorrhizal fungi exhibit host-dependent expression of secreted proteins that alters symbiotic efficiency in *Medicago truncatula*, *Nicotiana benthamiana* and *Allium schoenoprasum* [24]. The fact that such transcriptional variability has been linked to success of generalist pathogens led us to investigate the hypothesis that polyphagous plant-parasitic nematodes such as *P*. *coffeae* also have this ability.

RNA interference (RNAi) has been used to show that cell wall degrading enzymes have important roles in root penetration by migratory nematode species [17, 25, 26]. The inability to degrade one or more cell wall components, such as cellulose or xylan, results in failure to enter the root and death of the nematode. Consequently, as cell wall composition is known to vary between plant families, we focused on determining if expression of these enzymes in *P*. *coffeae* is modulated in response to different host species. Substantial differential expression of both a β-1,4-endoglucanase (*Pc-eng-1*) gene and a β-1,4-endoxylanase (*Pc-xyl*) gene occurred in *P*. *coffeae* post-host invasion and in response to exposure to exudates from host roots. Importantly, the magnitude of this response differed significantly between hosts. A positive correlation occurred between expression levels of these two genes and the level of cellulose and xylan in root exudates. RNAi-mediated loss of transcripts of either gene reduced establishment of this nematode in both potato and maize roots, thereby indicating that the correct regulation of these genes is important for parasitism.

## Results

### Host root exudates stimulate a stylet thrusting response

**Fig 1:**
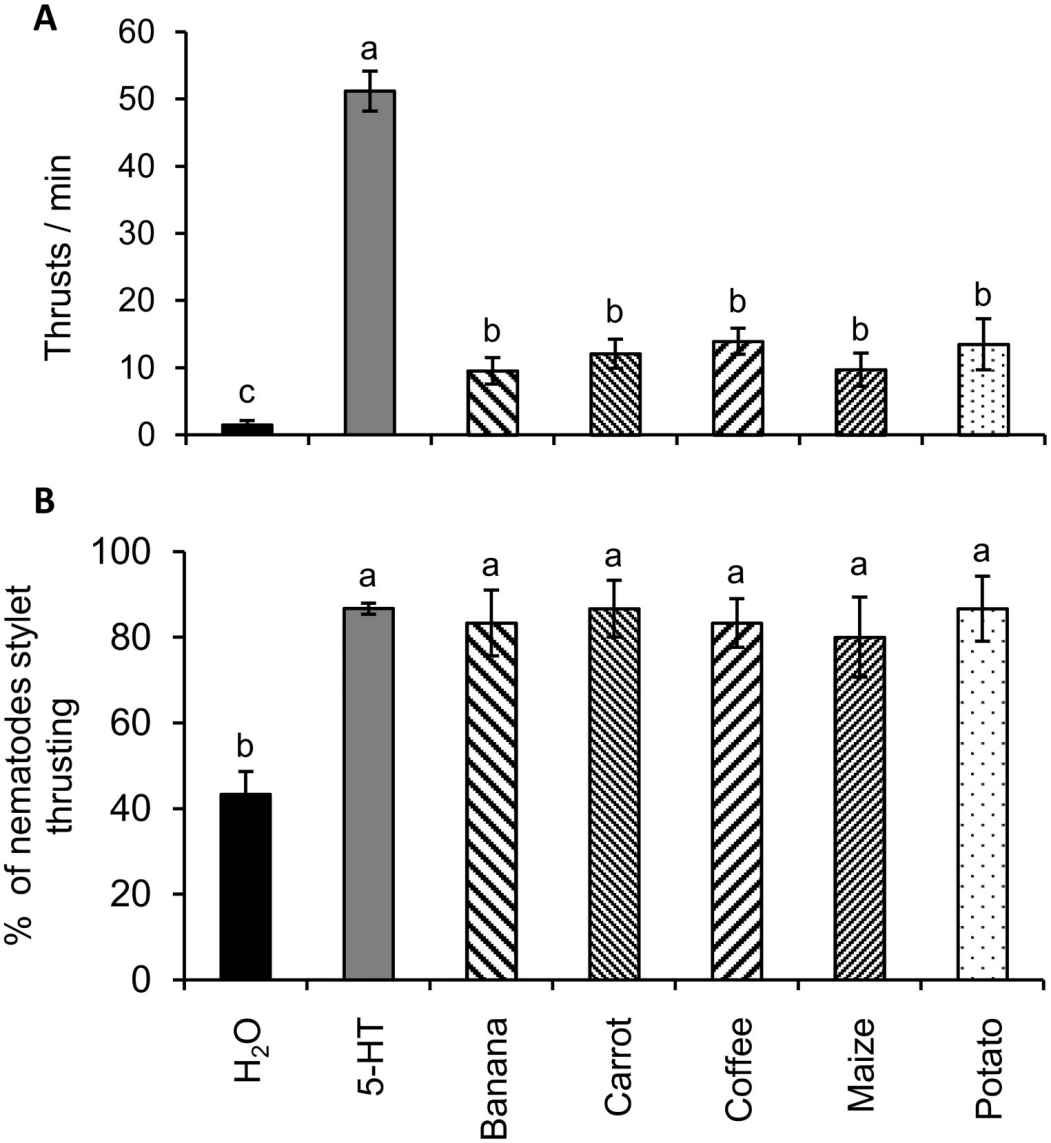
Host root exudates stimulate a stylet thrusting response in *P*. *coffeae*. Root exudates from the hosts banana, carrot, coffee, maize and potato were evaluated for their effects on the rate of stylet thrusting in individuals of mixed life stages (A) and the proportion of individuals of mixed life stages thrusting their stylets (B). H_2_O and 5 mM 5-hydroxytryptamine (5-HT) were used as negative and positive controls, respectively. Values are means ± SEM (n = 30 with different letters indicating significant differences between treatments P<0.01 (One-way ANOVA, SNK test).

Exposure to root exudate is known to stimulate thrusting of the stylet in some plant parasitic nematodes [20] although the response of *Pratylenchus* spp. has not been reported. *Pratylenchus coffeae* has a wide host range encompassing both monocot and dicot plants from >30 different genera [14]. We therefore first tested if root exudates from five representative monocot and dicot plants from its host spectrum (coffee, banana, maize, carrot and potato) induce stylet thrusting. Incubation in root exudates of all five hosts stimulated a significant increase in stylet thrusting of mixed stages of *P*. *coffeae* by 6–9 fold or more relative to a very low frequency in water (Fig 1A). The proportion of nematodes showing any frequency of stylet thrusting also significantly increased and almost doubled for all root exudate treatments relative to water (Fig 1B; One-way ANOVA, SNK, P<0.001). All root exudates induced the same response regardless of plant identity. As a positive control, *P*. *coffeae* were incubated in 5 mM 5-hydroxytryptamine (5-HT), which is known to stimulate stylet thrusting in plant parasitic nematodes, including the related species *Pratylenchus penetrans* [27]. Exposure to this concentration of 5-HT induced an even higher rate of thrusting (Fig 1A; One-way ANOVA, SNK, P<0.001) but not a significant, further increase in the high proportion of nematodes responding to root exudate (Fig 1B).

### *Pc-eng-1* and *Pc-xyl* are expressed in the pharyngeal gland cells throughout nematode development

**Fig 2:**
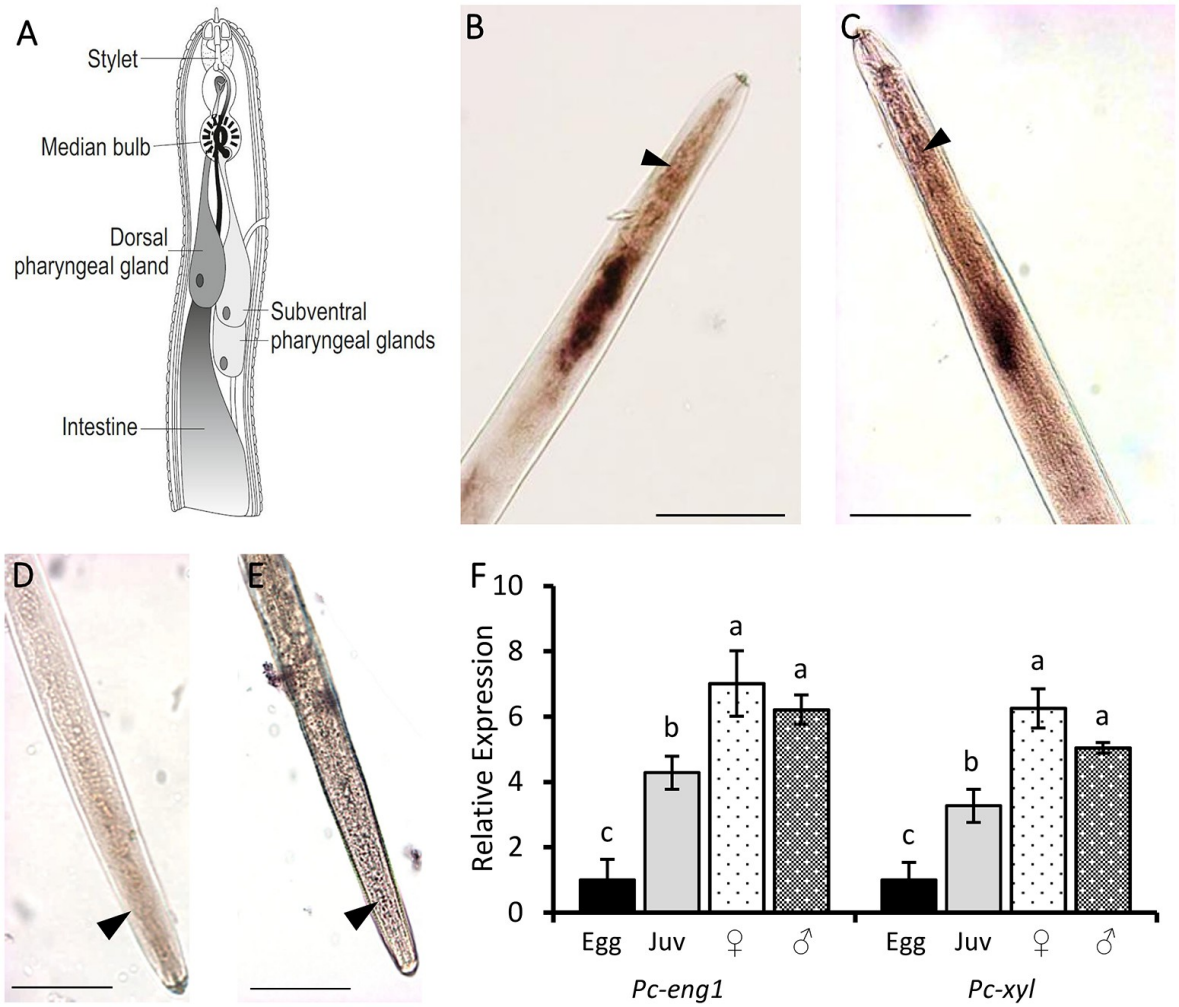
*Pc-eng-1* and *Pc-xyl* are expressed in the pharyngeal gland cells throughout nematode development. Schematic representation of a plant-parasitic nematode (A) outlines the pharyngeal gland cells where the digoxigenin-labelled probes of *Pc-eng-1* (B) and *Pc-xyl* (C) are localised. The median bulb is indicated for reference (arrow). No corresponding staining occurred when the negative control probes were used (D and E). Scale = 50 μm. Analysis by qRT-PCR confirmed that *Pc-eng-1* and *Pc-xyl* are both expressed at egg, juvenile (juv), female and male life stages (F). Both genes showed significantly increased expression as the nematode developed. Expression was normalised to Elongation Factor and presented relative to expression in eggs. Values are means ± SEM (n = 3 pools of individuals) with different letters indicating significant differences between treatments P<0.05 (One-way ANOVA, SNK test).

Nematode stylet activity is an essential component for both invasion of and migration through roots. In order to determine if exposure to host root exudate also affected expression of *P*. *coffeae* genes involved in the invasion and migration process, we first characterised the spatial and temporal expression of two genes that encode plant cell wall degrading enzymes. A xylanase-encoding sequence (*Pc-xyl*) was identified by homology searching following *de novo* assembly of publicly available transcript data for *P*. *coffeae*. Expression of both this and a previously reported β-1,4-endoglucanase gene [16] (*Pc-eng-1*) were localised by *in situ* hybridisation to the secretory pharyngeal glands of mixed life stages (Fig 2B and 2C). No corresponding staining occurred when the negative control probes were used (Fig 2D and 2E).

Analysis by qRT-PCR confirmed that *Pc-eng-1* and *Pc-xyl* are both expressed by all the nematode life stages studied. Both genes showed significantly increased expression as the nematode developed on carrot discs. The fold increase of *Pc-eng-1* transcript for combined adult males and females relative to eggs was 6.22 ± 0.63 and 4.29 ± 1.11 for juveniles (Fig 2F). The pattern of expression for *Pc-xyl* was similar to that for *Pc-eng-1* (Fig 2F). The level of expression did not differ significantly between males and females for either gene.

### Expression of *Pc-eng-1* and *Pc-xyl* is influenced by the host plant

**Fig 3:**
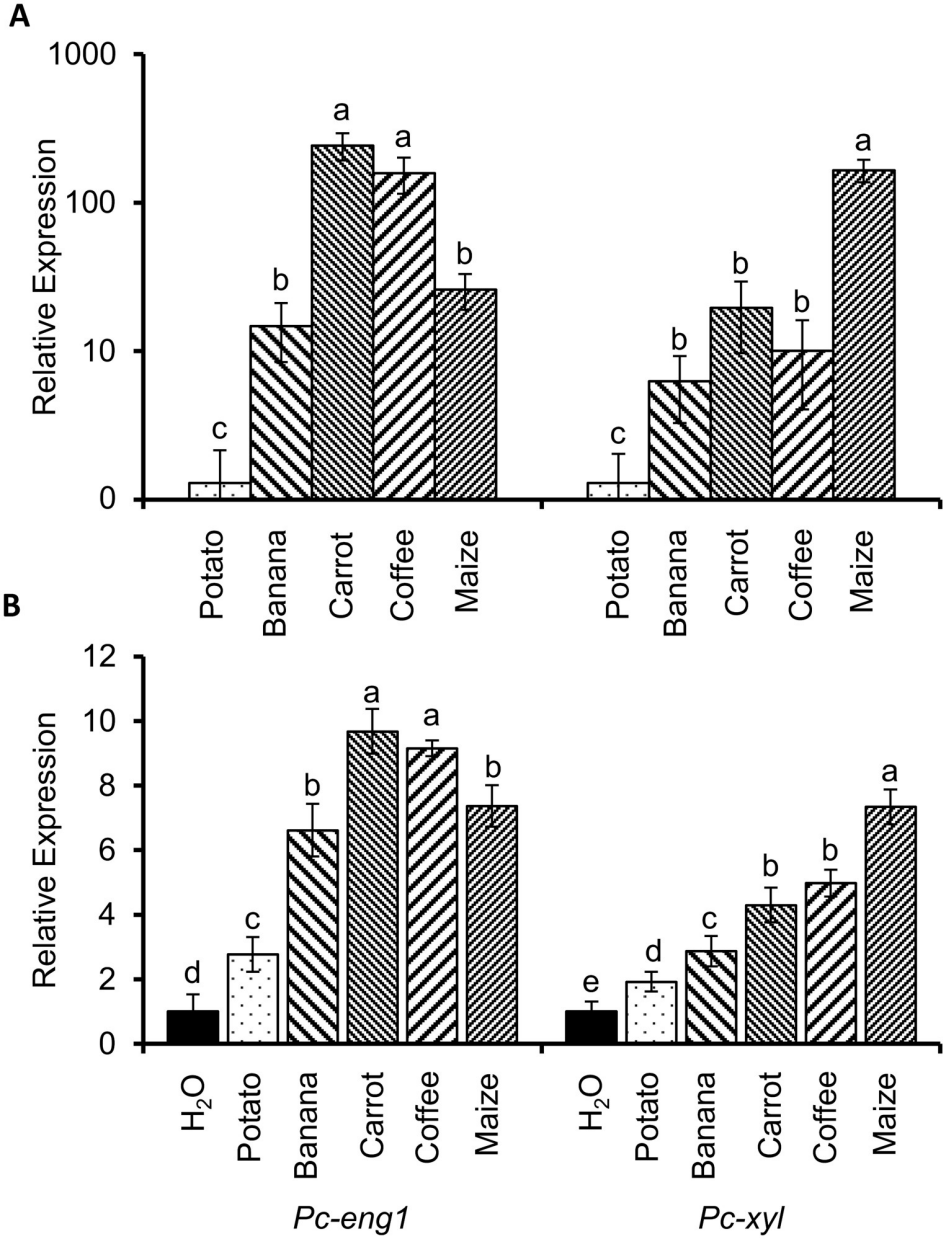
Expression of *Pc-eng-1* and *Pc-xyl* is influenced by the host plant. Relative expression of *Pc-eng-1* and *Pc-xyl* significantly differed between mixed life stage pools of *P*. *coffeae* recovered from host roots of banana, carrot, coffee, maize or potato (A). Values are means ± SEM (n = 8 pools of mixed stages) shown relative to expression in nematodes recovered from the roots of potato plants. Exposure of nematodes extracted from the same host (carrot) to different root exudates after 48 h in water induced expression of *Pc-eng-1* and *Pc-xyl*. The expression profiles were broadly similar to those observed when nematodes were extracted directly from the different host roots. (B). Values are means ± SEM (n = 4 pools of mixed stages) shown relative to expression in nematodes maintained in water. Different letters indicate significant differences between treatments P<0.05 (One-way ANOVA, SNK test).

We next tested if the host plant species influenced the expression of the two genes. Microscopic examination of nematode pools prior to RNA extraction revealed no gross differences in the relative abundance of life stages recovered from the different host roots. These populations of mixed life stages of *P*. *coffeae* showed significantly higher expression of *Pc-eng-1* and *Pc-xyl* when extracted from roots of banana, carrot, coffee and maize than from roots of potato. Additional differences in expression were associated with host identity. Relative to nematodes from roots of potato, *Pc-eng-1* expression was higher when recovered from banana and maize (14.76 ± 6.34 fold, 26.06 ± 7.09 fold) and highest when recovered from roots of carrot and coffee (242.86 ± 50.01 fold, 158.19 ± 43.02 fold) (Fig 3A). *Pc-xyl* expression for nematodes parasitising roots of maize was 165.09 ± 28.75 fold that for individuals from roots of potato. This increase was greater than the grand mean increase in *Pc-xyl* expression for individuals extracted from banana, carrot and coffee (11.76 ± 3.67 fold) compared to nematodes from roots of potato.

Differential induction of gene expression was not dependent on contact of the nematodes with host roots. Exposure of nematodes cultured on the same host (carrot) to different root exudates after 48 h in water induced broadly similar expression profiles of *Pc-eng-1* and *Pc-xyl* as root parasitism. *Pc-eng-1* expression in mixed life stages of *P*. *coffeae* was significantly higher after incubation in any of the host exudates than in water (Fig 3B). The magnitude of this increased expression was highest for nematodes exposed to exudates of carrot or coffee. Those values were greater than for individuals in either banana or maize exudates which in turn were significantly higher than that for nematodes exposed to potato root exudate (Fig 3B). This pattern of relative expression mirrored that observed for *Pc-eng-1* when nematodes were extracted from the different host roots. The expression of *Pc-xyl* was also significantly higher for nematodes incubated in root exudates than water but the pattern of expression among host exudates did not match that for *Pc-eng-1*. Expression of *Pc-xyl* was highest for *P*. *coffeae* in maize exudate, similar but less upregulated for those in carrot or coffee exudates, lower when incubated in that of banana and least for nematodes in potato root exudate. This lowest level of induction of *Pc-xyl* by a host root exudate was still higher than for nematodes incubated in water only (Fig 3B).

### *Pc-eng-1* and *Pc-xyl* expression correlates with cellulose and xylan quantities exuded by plant roots

**Fig 4:**
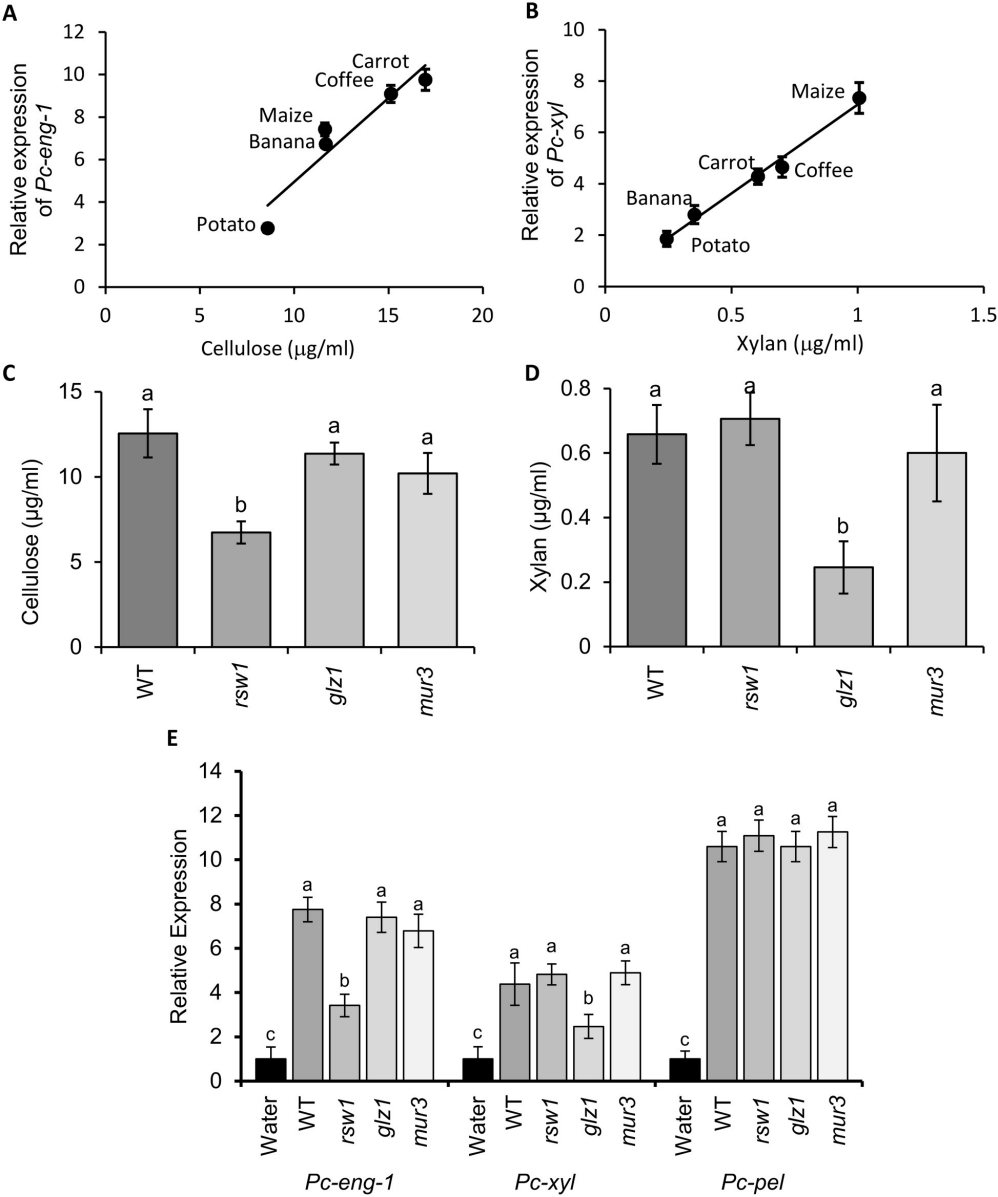
*Pc-eng-1* and *Pc-xyl* expression correlates with cellulose and xylan quantities exuded by plant roots. There was a linear regression between the quantity of cellulose and xylan in the exudates (n = 5) and the expression level of *Pc-eng-1* (A) (P<0.05, R^2^ = 0.89) and *Pc-xyl* (B) (P<0.01. R^2^ = 0.988) (n = 4 pools of mixed stages), respectively. Exudates from the roots of *Arabidopsis rsw1* and *glz1* were confirmed to be deficient in cellulose and xylan, respectively compared to Col-0 (WT) (C and D). Cellulose and xylan deficiencies caused significantly lower induction of *Pc-eng-1* and *Pc-xyl* expression in *P*. *coffeae* after 6 h exposure (E). *Pc-pel* remained unaffected by the changes in exudate whilst exudates from the roots of *mur3* had no effect on expression of any gene studied. Values are means ± SEM (n = 4 pools of mixed stage *P*. *coffeae*) with different letters indicating significant differences between treatments P<0.05 (One-way ANOVA, SNK test). Gene expression levels (A, B and E) are presented relative to expression in water.

We hypothesised that the host-related expression levels of *Pc-eng-1* and *Pc-xyl* might reflect relevant differences in root exudate composition. Therefore cellulose (substrate for β-1,4-endoglucanase) and xylan (substrate for β-1,4-endoxylanase) were quantified in each exudate. Overall there was a linear regression between the quantity of cellulose in the exudates and the expression level of *Pc-eng-1* (P<0.05, R^2^ = 0.89; Fig 4A). A linear relationship was also evident in the parallel experiment with increasing amounts of xylan inducing significantly increased expression of *Pc-xyl* (P<0.01, R^2^ = 0.988; Fig 4B). The rank correlation coefficient (Spearman test) for the comparison of the increased expression of the two genes in response to plant species was not significant because carrot and maize exudates had different ranks for concentration of the two substrates (Fig 4A and 4B).

Mutant *Arabidopsis* plants deficient in either cellulose or xylan were used to provide further evidence that concentrations of these cell wall components in root exudate specifically regulate expression of *Pc-eng-1* and *Pc-xyl*. Initial analysis confirmed that the reported cellulose deficiency of *rsw1* mutant plant tissue [28] and the xylan deficiency of *glz1* plants [29] was reflected in reduced accumulation of these molecules in root exudates (Fig 4C and 4D). Transcript abundance of both *Pc-eng-1* and *Pc-xyl* increased relative to expression in water after exposure of nematodes to root exudate from wildtype *Arabidopsis* plants (Fig 4E). Expression of *Pc-eng-1* was only reduced significantly (P <0.01; One-way ANOVA) from this level when the nematodes were exposed to exudates of the *rsw1* mutant line that is deficient in cellulose. A similar specific effect was obtained for *Pc-xyl* expression when the root exudate was obtained from *glz1* mutant plants deficient in xylan (P <0.01; One-way ANOVA). The specificity of the response was confirmed by analysing a pectate lyase gene (*Pc-pel*) and an additional *Arabidopsis* mutant (*mur3*). *Pc-pel* does not degrade or modify cellulose or xylan and was therefore predicted to be unresponsive to varying abundance of these two polysaccharides. The expression of *Pc-pel* was upregulated in response to *Arabidopsis* root exudate but no differential responses were detected among the exudates from wildtype and mutant plants. The *mur3* mutant is deficient in fucose and galactose sidechains on the hemicellulose xyloglucan, thereby providing no changes to cellulose or xylan abundance but altering the cell wall structure. The expression of all three nematode genes studied was unaffected, compared to wildtype, when the exudate was from roots of *mur3* plants.

### Cellulose and xylan specifically upregulate expression of *Pc-eng-1* and *Pc-xyl*, respectively

**Fig 5:**
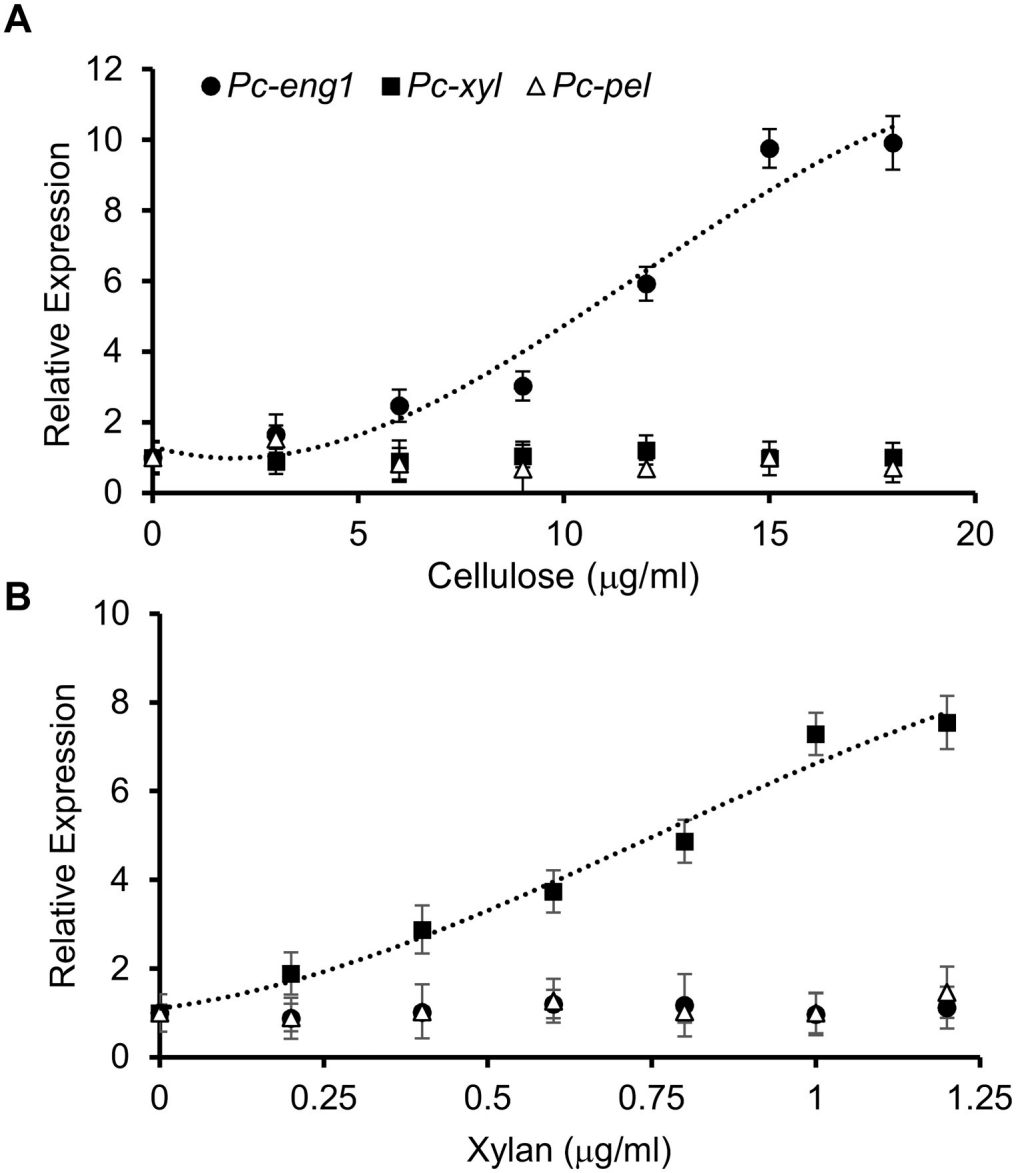
Cellulose and xylan specifically upregulate expression of *Pc-eng-1* and *Pc-xyl*, respectively. Polynomial regression analysis establishes a significant increase in expression of *Pc-eng-1* (black circle) and *Pc-xyl* (black square) in *P*. *coffeae* with cellulose (A) and xylan (B) concentrations respectively after 6 h exposure (P< 0.01, R^2^ = 0.96 and P<0.001, R^2^ = 0.98, respectively). Expression of *Pc-pel* (white triangle) remained unaffected. Values are means ± SEM (n = 4 pools of mixed stage *P*. *coffeae*) and relative to expression in water.

We next determined if pure solutions of cellulose or xylan could induce expression of the nematode genes. Exposure of batches of mixed stages of *P*. *coffeae* to a range of cellulose solutions resulted in a significant linear increase in expression of *Pc-eng-1* but not *Pc-xyl* or *Pc-pel* (Fig 5; P<0.05, R^2^ = 0.975). A parallel experiment with a range of xylan concentrations resulted in a linear increase in *Pc-xyl* expression (P<0.05, R^2^ = 0.955) but not of the other two genes.

### RNAi of *Pc-eng-1* and *Pc-xyl* reduces root invasion by *P*. *coffeae*

**Fig 6:**
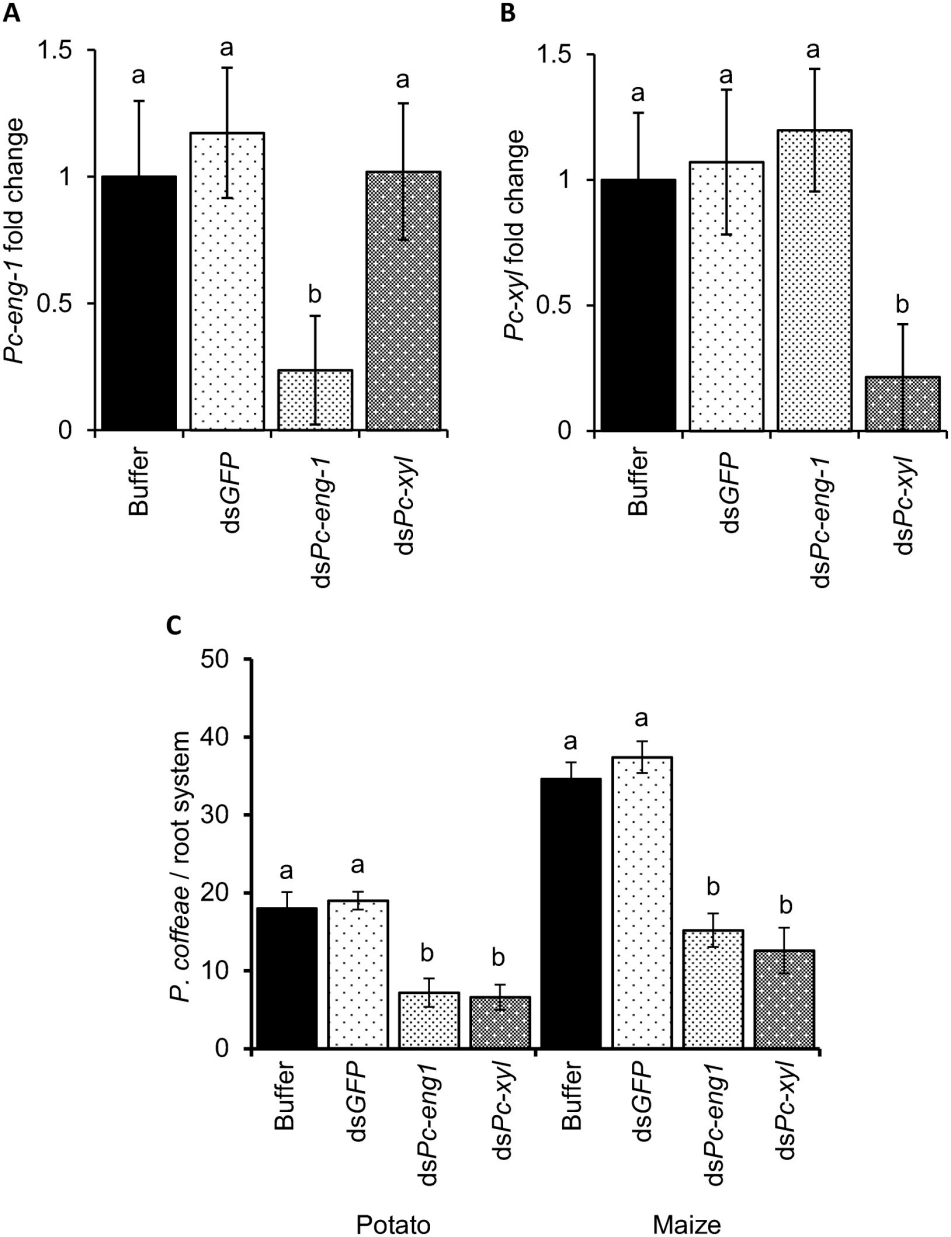
RNA interference of *Pc-eng-1* and *Pc-xyl* in *P*. *coffeae* reduces infection of potato and maize roots. The dsRNA molecules reduced expression of their respective targets whilst *gfp* dsRNA (a gene that is absent from the nematode) had no effect (A and B). Expression is presented relative to that for control nematodes incubated in buffer only. *Pc-eng-1* dsRNA and *Pc-xyl* dsRNA reduced the infection of *P*. *coffeae* in both potato and maize root systems (C). Values are means ± SEM (n = 6 pools of mixed stage *P*. *coffeae*) with different letters denoting significant differences between treatments P<0.05 (One-way ANOVA, SNK test).

RNA interference was used to establish if the induced expression of the *P*. *coffeae* endoglucanase and endoxylanase genes was important for successful invasion of host roots (Fig 6). Two different hosts were tested. Potato was selected as its exudates contain the least cellulose and xylan whilst maize exudate has the highest xylan content. In the absence of RNAi, maize roots proved to be the more readily invaded host; a significantly greater number of nematodes were present in maize than potato roots after allowing a 72h period for root invasion (Fig 6) (P <0.01; One-way ANOVA). Treatment of mixed stages of *P*. *coffeae* with a dsRNA solution specifically targeting *Pc-eng-1* reduced expression of this gene by 76% (Fig 6A). The number of dsRNA-treated *P*. *coffeae* detected in maize or potato plants after access to their roots for 72h was reduced significantly by 62.4 ± 5.1 and 54.4 ± 4.8% respectively relative to those nematodes pre-incubated in buffer only (Fig 6C) (P<0.001 in both cases; SNK, One-way ANOVA). A dsRNA targeting *Pc-xyl* reduced expression of this gene by 79% (Fig 6B). Targeting this gene reduced nematode numbers in maize and potato roots by 68.2 ± 6.1 and 41.1 ± 6.7% respectively (Fig 6C) (P<0.001 in both cases; SNK, One-way ANOVA). A control dsRNA treatment that targeted a *gfp* sequence not present in the nematodes was without effect on root invasion or expression of either gene.

## Discussion

The results establish that expression of two genes encoding cell wall modifying enzymes is upregulated in *P*. *coffeae* both post-invasion of roots and post-exposure to exudates from host roots. Furthermore, we demonstrate that this is a host-specific response; exposure to root exudates from different host plants confers a differential gene response in this plant-parasitic nematode. The five host plants tested could be divided into three significant groups with respect to their induction of expression of the β-1,4-endoglucanase gene *Pc-eng-1*: (coffee and carrot) > (maize and banana) > potato. These groupings were the same for expression of *Pc-eng-1* in nematodes recovered from the host roots and nematodes exposed to the root exudates. Three groups were also established for *Pc-xyl* expression but with a different rank of maize > (banana, carrot and coffee) > potato. The host-specific abundance of both transcripts in response to root exudate was linearly related to the level of cellulose and xylan respectively with potato exudates having the lowest concentrations of both complex carbohydrates. The responses were established as specific as the expression of *Pc-eng-1* increased with the concentration of cellulose but not xylan with a *vice versa* effect when *Pc-xyl* expression was measured. The expression of a pectate lyase (*Pc-pel)* was unaltered by exposure to either of these two complex carbohydrates, presumably due to the gene-product exhibiting no activity on either substrate. Pectate lyase catalyses the cleavage of unmethylated pectin and the nematode enzymes are predicted to aid in softening of the cell wall middle lamella so facilitating migration [30]. This gene does respond to presence of a host and so may be regulated by other specific or general components of root exudates. Determining whether or not individual exudate components have specific or broad effects on nematode gene expression is relevant to understanding the mechanism behind the response in not just *P*. *coffeae* but other plant-parasitic nematodes that upregulate genes in response to root exudates [20]. There is no indication that these enzymes are released in sequential order by the nematode, as observed in fungi which usually secrete pectin degrading enzymes first [31].

Use of mutant lines of *A*. *thaliana*, which is also a host for *P*. *coffeae* [32, 33], confirmed the specificity of the effect as expression of *Pc-eng-1* was reduced only when the nematode was exposed to exudate from a mutant deficient in cellulose [34] and *Pc- xyl* only when exposed to exudate from a mutant deficient in xylan [29]. Tissue from these mutants contains <50% of cellulose and xylan wild-type levels respectively [34, 35]. A third mutant deficient in fucose and galactose sidechains of xyloglucan had no effect on the expression level of either gene, confirming that a variation in cell wall composition *per se* does not necessarily incur changes in expression in the nematode. Although Arabidopsis is a host, we and others have found that the rate of infection and reproduction of *P*. *coffeae* in Arabidopsis is unreliable and highly variable [33]. Concerns about potential pleiotropic effects of Arabidopsis cell wall mutants further discouraged inquiry regarding the capacity of *P*. *coffeae* to invade and reproduce in the mutant lines. The use of root exudates from additional mutant lines, coupled with a panel of nematode genes, provides the opportunity to further analyse exudate components that elicit specific or broad responses in the nematode. RNAi of either *Pc-eng-1* or *Pc-xyl* reduced nematode invasion of maize and potato roots and established the importance of both these gene products for the penetration of both hosts.

The polysaccharide components of exudates form a mucilaginous layer along the root, often accumulating at the root tip [1]. These components are either secreted by root epidermal cells or released through their degradation [2, 3]. Transfer of polysaccharides across the cell membrane has been suggested to occur through vesicular trafficking and/or ATP-binding cassette transporter proteins [3, 36]. Although costly for the plant, continual communication with the rhizosphere is considered important in order to detect and respond to the presence of pathogens, symbionts and beneficial soil micro-organisms [1]. However, in this study we found that these compounds may also be reliable indicators of close proximity of roots and so induce preparation of *P*. *coffeae* for root invasion. Expression of *Pc-eng-1* and *Pc-xyl* by all life stages studied is also appropriate as all stages of this nematode invade roots during development. The host-specific concentration of cellulose and xylan is detected by the nematode resulting in appropriate transcriptional shifts. This distinctive reaction to non-host specific complex carbohydrates may be an adaptation for plant invasion due to the polyphagous nature of *P*. *coffeae*. Transcriptional plasticity in response to different plant hosts is known to occur for the generalist aphid *Myzus persicae* [23] and may also be important for other polyphagous plant-feeding arthropods [37]. Our results extend this response to a generalist plant-feeding nematode, suggesting it may be a common adaptation to tailor gene expression to a particular host plant. It would be interesting to investigate which nematode species and genera are capable of such perception and determine if this relates to host generalisation rather than specialisation. Mitotic asexual species of *Meloidogyne*, root-knot nematodes, which cause complex adaptive changes in root cells to form feeding sites, also have wide host ranges. Their polyphagy is considered to relate to the plasticity afforded by their large, duplicated genomes [38]. This approach is an interesting contrast with that of *Pratylenchus*. This genus does not modify plant cells but achieves a wide host range while having the smallest genome of any nematode studied to date [39]. Attempting to establish in any plant root encountered seems to be a beneficial adaptation given the nematode’s limited locomotory range in soil. The optimised expression of *Pc-eng-1* and *Pc-xyl* seems likely to contribute to the success of generalist *P*. *coffeae* nematodes although environment-adjusted regulation of these genes may not play a dominant role in determining the actual host status of different plant types. Root invasion rates and subsequent success in feeding and reproduction are influenced by many factors associated with both the root and the nematode. The recent sequencing of the *P*. *coffeae* genome may enable genes involved in those aspects of the host/parasite interaction to be defined [39].

Direct chemoreception of carbohydrate polymers is not a commonly reported ability. Intracellular fluctuations in transcription factor binding due to external cellulose or xylan have been reported for filamentous fungi [40]. Transcription factors CLR-1 and XLR-1 in *Neurospora crassa* bind to promoters of genes encoding cellulases and xylanases, respectively, with binding enrichment observed for both when grown in cellulose or xylan conditions [41]. Similar responses may occur in plant-parasitic nematodes that regulate the differential expression of cell wall-degrading enzyme genes. However, it is unclear from the fungal studies whether or not the polymer itself is detected, or if the observed effects occur in response to the presence of breakdown products.

Plants can perceive cellulose-derived oligomers as damage-associated molecular patterns (DAMPs) as a means to survey cell wall integrity and then respond by activating a signalling cascade that leads to induction of defence-related genes [42]. Monosaccharides are also known to induce the upregulation of several genes, including an endoxylanase, in fungi [43, 44]. Chemosensory detection of these breakdown products by *P*. *coffeae* in root exudates may result in the host-specific expression of *Pc-eng-1* and *Pc-xyl*. Monosaccharides are present in root exudates [45] and influence nematode chemo-attraction and stylet activity [46]. In the field, such breakdown products could arise from the activities of soil microbes or be generated in proximity to the nematode through the action of its own secreted enzymes. Given that sterile solutions of cellulose and xylan elicited similar induction of gene expression as root exudate containing equivalent concentrations, it seems likely that soil microbes are not playing an important role in this case. Both *Pc-eng-1* and *Pc-xyl* were expressed at detectable basal levels when *P*. *coffeae* was maintained in water and the nematodes exhibited a low rate of stylet thrusting in these conditions. This activity might supply sufficient amounts of the enzymes to release soluble inducers from the carbohydrate polymers associated with the roots, as proposed for fungi [47]. As for other typical β-1,4-endoglucanases, *Meloidogyne incognita* ENG-1 has been shown to cleave cellulose into glucose dimers/trimers rather than monosaccharides [48]. The conservation of GH5 cellulases suggests that the enzymes of other Clade 12 nematode species, such as *P*. *coffeae*, likely have similar activity [49]. The breakdown of these small glucose chains requires β-glucosidase, which we have not identified as being encoded in the genome or transcriptome data for *P*. *coffeae*. This suggests a system based on detection of either the cellulose polysaccharide or the short chain cellobiose/cellotriose breakdown products, rather than the monosaccharides. A parallel effect is known for filamentous fungi which do not require the breakdown of oligosaccharides into glucose monomers for induction of β-1, 4-endoglucanase genes [50]. These data present new insights into pathogen detection of carbohydrate polymers and its importance in the parasitism of an economically important nematode species.

## Methods

### Plant culture and exudate collection

Banana (*Musa acuminata*), coffee (*Coffea arabica*) and maize (*Zea mays*) plants were grown in 50:50 sand/loam mix in a glasshouse at 23–25 °C with supplementary lighting to provide 16:8 h light:dark conditions. Carrot (*Daucus carota*) and potato (*Solanum tuberosum* var. Désirée) were grown similarly at 19–22 °C. *Arabidopsis thaliana* (Col-0, *rsw1-1* (NASC ID: N6554), *glz1* (NASC ID: N16279) and *mur3* (NASC ID: N8566)) were grown at 28 °C on ½ strength Murashige and Skoog medium containing 1% sucrose. For exudate collection, roots were washed, separated intact from above ground tissue and soaked in water (80 g/L) in darkness for 24 h at 4 °C. Root exudates were then filter sterilised (0.22 μm) and stored at 4 °C. For RNAi experiments maize and potato plants were grown in CYG growth pouches (Mega International, USA) for 8 days at 22°C before infection.

### Nematode culture and plant infection

A population of *P*. *coffeae* was maintained on sterile carrot discs at 26 °C for use in assays described below and to provide inoculum for infection of different host plants. Mixed life stages were collected by washing the discs with sterile tap water. Batches of 500 nematodes were introduced into the soil to a depth of 2 cm around the stem of each host plant for infection. Roots were harvested after eight weeks and washed to remove soil. Roots were then immediately placed in a misting chamber where the spray of water stimulated the movement of nematodes out of the root [51]. After six hours the nematodes were collected in water from the chamber.

### Stylet thrusting assay

Groups of 100 mixed life stages *P*. *coffeae* nematodes collected from carrot discs were soaked in either 100 μl sterile water, 5 mM 5-hydroxytryptamine (5-HT) or a root exudate for 1 h [20]. Ten nematodes were observed at a magnification of 250x and stylet thrusts of each nematode were counted for 30 s in triplicate. This protocol was replicated with three independent exudates to account for possible variation between collections.

### Gene expression analysis

#### Nematodes extracted from roots

Nematodes were collected from the roots of banana, carrot, coffee, maize and potato plants using a misting chamber, as described above. Nematodes were frozen and total RNA was then extracted, as described later. This was replicated eight times for nematodes from each host plant.

#### Post-exposure to host root exudates

Mixed stage nematodes of *P*. *coffeae* were removed from carrot disc cultures and washed multiple times. Samples of 500 mixed stage nematodes were incubated in tap water for 48 h before exposure to 500 μl of either host root exudate or fresh tap water for 6 h. Total RNA was extracted immediately, as described later. This was carried out four times for each treatment.

#### Throughout nematode development

Groups of 100 eggs, juveniles, females and males were selected individually from a mixed nematode population reared on carrot discs, and total RNA was extracted to determine expression of genes at different nematode life stages. This was repeated in triplicate.

#### Treatment of nematodes with cellulose and xylan

Sets of 500 mixed stage *P*. *coffeae* were removed from carrot disc cultures and washed multiple times before incubating in tap water for 48 h. The nematodes were then treated with a range of cellulose (0–18 μg/ml) or xylan (0–1.2 μg/ml) solutions (Sigma-Aldrich, US) for 6 h. Concentration ranges were chosen based on respective polysaccharide concentrations detected in root exudates, as described later. This was carried out four times per concentration treatment and then total RNA was extracted.

### RNA extraction, cDNA synthesis and gene expression analysis

Total RNA was prepared from nematode samples using an RNeasy Plant Mini Kit according to the manufacturer’s protocol including DNase treatment (Qiagen, UK). First-strand cDNA was synthesised from 750 ng RNA using SuperScript II reverse transcriptase (Invitrogen, UK) and Oligo(dT)_17_ primer (500 μg/ml) following the manufacturer’s protocol. Analysis of gene expression was carried out using quantitative reverse transcription (qRT) PCR with Brilliant III Ultra-Fast SYBR Green Master Mix (Agilent Technologies, CA, USA). Cycle conditions were 95 °C for 30 s and subsequently 40 cycles of 5 s at 95 °C and 10 s at 60 °C. The sequence of *Pc-eng-1* was obtained from GenBank (EU176871.1 [16]), whereas *Pc-xyl* (endoxylanase), *Pc-pel* (pectate lyase) and *Pc-ef* (elongation factor, a reference gene) sequences were obtained from genome and transcriptome sequence reads (PRJNA276478 [39], PRJNA79895 [52]) that were assembled using GS *De Novo* Assembler Software (Roche) (sequence data is given in S1). The genomic data were used to identify regions of *Pc-eng-1*, *Pc-xyl*, *Pc-pel* and *Pc-ef* that were suitable for design of sequence-specific qRT-PCR primers (see S2 for primer sequences). Each primer pair had an amplification efficiency of 95–100%. The expression of *Pc-ef* was confirmed to be stable across treatments and life stages, validating its use as a reference gene (S3). The 2^(-ΔΔCt)^ method was used to calculate relative expression between control and experimental samples for at least three biological replicates each with three technical replicates. One-way ANOVA with a Student-Newman-Keuls *post hoc* test was used to determine significant differences between means unless otherwise stated.

### *In situ* hybridisation

Single-strand digoxygenin-labelled anti-sense DNA probes for *Pc-eng-1* and *Pc-xyl* were synthesised with DIG DNA labelling mix (Roche, Germany) from cDNA fragments amplified by qPCR primers for *Pc-eng-1* and *Pc-xyl* (see S2 for primer sequences). Sense probe controls were constructed in separate reactions. These probes were used for *in situ* hybridisation to determine spatial expression patterns for both genes [53]. Approximately 2000 *P*. *coffeae* were fixed in 2% paraformaldehyde in M9 buffer for 18 h at 4 °C followed by 4 h at 22 °C. Fixed nematodes were cut with a razor blade before washing with M9 buffer and proteinase-K treatment (0.5 mg/ml for 30 min at 22 °C). Nematodes were frozen and treated with methanol for 1 min followed by acetone for 1 min before rehydration in RNase-free water. Treated nematodes were hybridised with the probes overnight at 50 °C and then washed 3 times with 4x saline sodium citrate (SSC) and three times with 0.1x SSC/ 0.1% SDS at 50 °C. Nematodes were incubated at 22 °C in 1% blocking reagent in maleic acid buffer (Roche, Germany) for 30 min and labelled for 2 h with anti-digoxigenin-AP Fab fragments 1:1000 in 1% blocking reagent. The nematodes were stained overnight at 4 °C with 337 μg/ml nitroblue tetrazolium and 175 μg/ml 5-bromo-4-chloro-3-indolyl phosphate. Stained nematodes were washed in 0.01% Tween-20 before viewing under a compound microscope (Olympus, BH2). Images were captured with a QIcam camera (QImaging) and Q-Capture software.

### Quantification of cellulose and xylan in root exudates

Cellulose was quantified in root exudates by a colorimetric assay [54]. 1 ml of root exudates were centrifuged at 10 000 rpm for 5 min and supernatant removed. 0.3 ml acetic/nitric reagent (8:1:2, acetic acid:nitric acid:water) was added. Samples were incubated for 30 min at 90 °C and then centrifuged again before washing with 0.5 ml water. Samples were vortexed in 0.5 ml sulfuric acid (67%) before mixing with 1 ml of cold anthrone reagent (0.2% anthrone (Sigma-Aldrich, US) in sulfuric acid) and incubated at 90 °C for 16 min. Samples were then left to stand at 22 °C for 10 min before reading absorbance at 620nm. Five biological replicates were measured in technical triplicate and the optical densities were used to generate cellulose equivalents using a standard curve.

The antibody LM11 was used in an enzyme-linked immunosorbent assay to detect xylan in the root exudates [55]. Each exudate was incubated overnight at 4 °C with PBS (Severn Biotech) to ensure efficient coating of microtitre plate wells. Samples were diluted fivefold to ensure final absorbance readings in the range 0.1–1.0 optical density. Three technical and four biological replicates of each exudate were assayed. Plates were then blocked with 5% w/v milk powder (Marvel) in PBS before incubation with LM11 in 5% blocking solution at 22 °C for 1 h. LM11 was then incubated with the anti-HRP rat secondary antibody (A9552; Sigma-Aldrich, US) using a 1:1000 dilution. Substrate was added to each well (0.1 M sodium acetate buffer pH 6, 1% (v/v) tetramethyl benzidene, 0.006% (v/v) H_2_O_2_) to detect antibody binding, Five biological replicates were measured in technical triplicate and the optical densities were used to generate xylan equivalents using a standard curve.

### RNAi of *Pc-eng-1* and *Pc-xyl*

cDNA from *P*. *coffeae* was used to amplify templates for production of dsRNA complementary to *Pc-eng-1* and *Pc-xyl* (see S2 for primer sequences). The dsRNAs were designed to be sequence-specific using the assembled genome data to avoid suppression of non-target sequences. A GFP sequence [56] was amplified to provide a control for a non-nematode gene [57]. The DNA fragments were cloned between the XbaI and XhoI sites of the vector L4440 (pPD129.36 [58]). Complementary single-stranded RNAs (ssRNAs) were synthesised from T7 promoters in the L4440 constructs post-independent digestion with XbaI and XhoI. The synthesis of ssRNAs and subsequent production of double-stranded RNA (dsRNA) used a Megascript T7 RNAi kit (Invitrogen), according to the manufacturer’s instructions. A total of 500 mixed stage *P*. *coffeae* nematodes were treated with 100 μg/ml dsRNA in M9 buffer for 16 h at 25 °C. Control nematodes were treated with buffer only. Three-hundred individuals were used for RNA extraction and cDNA synthesis as described above to assess the reduction in target gene expression by qPCR. One hundred of the treated nematodes were infected on to roots of eight day old maize or potato plants grown in soil-free pouches, as previously described [59]. The 100 nematodes were distributed between five root tips on the root system. Each treatment was replicated six times. After 72 h, root tissue was stained with acid fuchsin [60] to visualise and count nematodes.

## Supporting information

S1 Fig Gene sequences. Nucleotide sequences for the genes identified in this study (*Pc-xyl*, *Pc-pel* and *Pc-ef*).
(DOCX)

S2 Fig Primer sequences used for analysis of selected sequences. Restriction sites for cloning are underlined.
(DOCX)

S3 Fig C_t_ values of *Pc-ef* expression in *P*. *coffeae* determined by qRT-PCR using equal quantities of cDNA of different life stages and post-treatment with different root exudates. Values are means ± SEM (n = 4 pools of mixed stages).
(DOCX)
