# Supplementary material for: Plant-parasitic nematodes respond to root exudate signals with host-specific gene expression patterns
Source: PLoS Pathog. 2019 Feb 1;15(2):e1007503. doi: 10.1371/journal.ppat.1007503 (PMC6373980; doi:10.1371/journal.ppat.1007503)
Supplement: S1 Fig — Nucleotide sequences for the genes identified in this study (Pc-xyl, Pc-pel and Pc-ef). (DOCX) [file ppat.1007503.s001.docx]

**S1 Fig: Gene sequences.** Nucleotide sequences for coding regions of the genes identified in this study (*Pc-xyl, Pc-pel* and *Pc-ef*).

>*Pc-xyl*

ATGACCGAACATTATAACGATGGCAACAGCTGGGCGAGCTGCatgCTGACCGCGAAAGAAATTCATGATAGCatgACCGTGGCGGATTATAACGCGTATCTGCTGTGGTGGTTTAAAGATGATACCAACCTGGGCCCGATTGATGGCAACGGCAACCCGACCatgCGTGGCTATGTGATTGGCCAGTGGGCGAAATATATTCGTCCGGGCTATGTGCGTGTGGATGCGACCTATAACCCGAGCAGCAACGTGTATGTGAGCGCGTATAAAAGCGGCAGCAAAGTGGTGATTGTGGCGGTGAACACCGGCGGCAGCGCGGTGAGCCAGCAGTTTAGCGTGAGCGGCGGCACCGTGCCGAGCAGCTTTACCCCGCATATTACCAGCAGCAGCAAAAAAATTAGCAACGAAGCGAACGTGAACGTGGCGAACGGCAGCTTTACCTATAGCCTGCAGGCGCAGAGCGTGACCACCTTTTGCGTGGAACTGAAAGAAGAAATTGAAGGCTGA

>*Pc-pel­*

ATGGCCACCATCGCAAATTGCATCATCGGCGCCAAATCGGGGACATCCGGCAGTTCGGATGGAATTCATTGCAAAGGCAGCTGCACACTCAAAAATGTCTGGTTTGAGGACGTGGGAGAAGATGCGGCCACATTTTATGGATTATCGTCGGACAGTCTAACCTACACGGTGGAGGGAGGAGGCGCCAAAAATGCGCAAGATAAAGTTTTCCAACACAATGGCAAGGGCACAGTTCGGATCAACAATTTTTGGGTGGACACATTCACTCGATTCTTTCGTTCCTGTGGAAATTGTGAGAACCAATATGCGCGCCATGTGGTCATCAAAAATTTGACGGCATTGAACGGGGTCAGCGGCCAATTCATTGCCGGCATTAATTTCAATTACGGAGACTCGGCAACACTCAGCCAAATAAAATTGGGCGGATCGACGGCGAAAAAaGTTGCCCCTTGCAAAAGATTCGTTGGGGTGACCAGCGgggAGAGCAAATCGAATGGGACGAATCCGGACGGAAAATATTGCATTTACAAAGAATCGGACATCACATATTTGTGA

>*Pc-ef*

AACATCGAACGCAAGGAGGGCAATGCAAGTGGCAAGACCCTGCTGGAAGCTTTGGACGCAATTGTTCCGCCGTCGAGGCCAACCGACAAGCCTTTGCGCCTTCCACTCCAGGATGTCTACAAGATTGGAGGTTTTCTTTAAAAATTGATAAACATTTTAATCATCATTCTATTGATTAGGTATTGGAACTGTGCCGGTCGGTCGTGTTGAAACTGGAGTGCTGAAGCCTGGAATGGTTGTGACGTTCGCACCACAAGGAATCTCCACTGAAGTGAAGTCCGTCGAAATGCATCACGAGTCCTTGCCAGAGGCCGTGCCTGGGGACAATGTTGGCTTTAACGTCAAGAACATCTCCGTGAAGGACATTCGTCGTGGCTCCGTTGCTTCCGACTCGAAAAaCGACCCAGCCAAGGAGGCCAAGCAATTCACCGCCcAGGGTAAAATTgAaTTTGATTtAAaGTTATGATTTTtACATGGATTTCAGTTATCATCATGAATCACCcGGGTCAAATCTCTGCCGGCTACACCCCGGTTCTGGACTGTCACACcGCCcACATTGCTTGCAAGTTTGCCGAACTCAAGGAGAAGGTCGACCGTCGTACTGGCAAGAAAGTTGAGGAcGcTCCCAAGGCACTCAAaAGcGGTGATGCAGGTTTGCTTTTgggatttGGgAATATTTaGTTTAGTCAATTAAAtttttttAGGCATtGTtGATTTGATTCCGTCGAAGCCCCTTTGCGTTGAGGCTTTCACCGACTACGCACCATTGGGACGTTTCGCCGTTCGGTAA
