# Supplementary material for: Plant-parasitic nematodes respond to root exudate signals with host-specific gene expression patterns
Source: PLoS Pathog. 2019 Feb 1;15(2):e1007503. doi: 10.1371/journal.ppat.1007503 (PMC6373980; doi:10.1371/journal.ppat.1007503)
Supplement: S2 Fig — Restriction sites for cloning are underlined. (DOCX) [file ppat.1007503.s002.docx]

S2 Fig: Primer sequences used. Restriction sites for cloning are underlined.

| Code | Purpose | Primer sequences |
| --- | --- | --- |
| *Pc-eng-1* | qPCR | F:Gctttggtgcaaaccgtcat  R:CACGTGATATGGCTTCACAACG |
| *Pc-eng-1i* | *in situ* | F:GCTCCCCCTTCTACACGGCC  R:TGGAGCCATATTGCCTGGCT |
| *Pc-xyl* | qPCR/ *in situ* | F:GCGTGTGGATGCGACCTATA R:CTGCTGGTAATATGCGGGGT |
| *Pc-pel* | qPCR | F:TTCCAACACAATGGCAAGGG R:CGCGCATATTGGTTCTCACA |
| *Pc-ef* | qPCR | F:CAAGGAGGCCAAGCAATTCAC R:AGAGATTTGACCCGGGTGATTC |
| *Pc-eng-1-RNAi* | RNAi | F:ATCGTCTAGAGCTCCCCCTTCTACACGGCC  R: ATCGCTCGATGGAGCCATATTGCCTGGCT |
| *Pc-xyl-RNAi* | RNAi | F:ATGCTCTAGAGATACCAACCTGGGCCCGAT R:ATGCCTCGAAGCAGCAGCAAAAAAATTAGCA |
| *RNAi-GFP* | RNAi | F:ATCGTCTAGAGCACTATTGCGGACTTGAAACA |
|  |  | R:ATCGCTCGAGCCATATTACGCGCTCCAGTT |
