# Supplementary material for: Plant-parasitic nematodes respond to root exudate signals with host-specific gene expression patterns
Source: PLoS Pathog. 2019 Feb 1;15(2):e1007503. doi: 10.1371/journal.ppat.1007503 (PMC6373980; doi:10.1371/journal.ppat.1007503)

**S3 Fig: C_t_ values of *Pc-ef* expression in *P. coffeae* determined by qRT-PCR using equal quantities of cDNA of different life stages and post-treatment with different root exudates.** Values are means ± SEM (n= 4 pools of mixed stages).


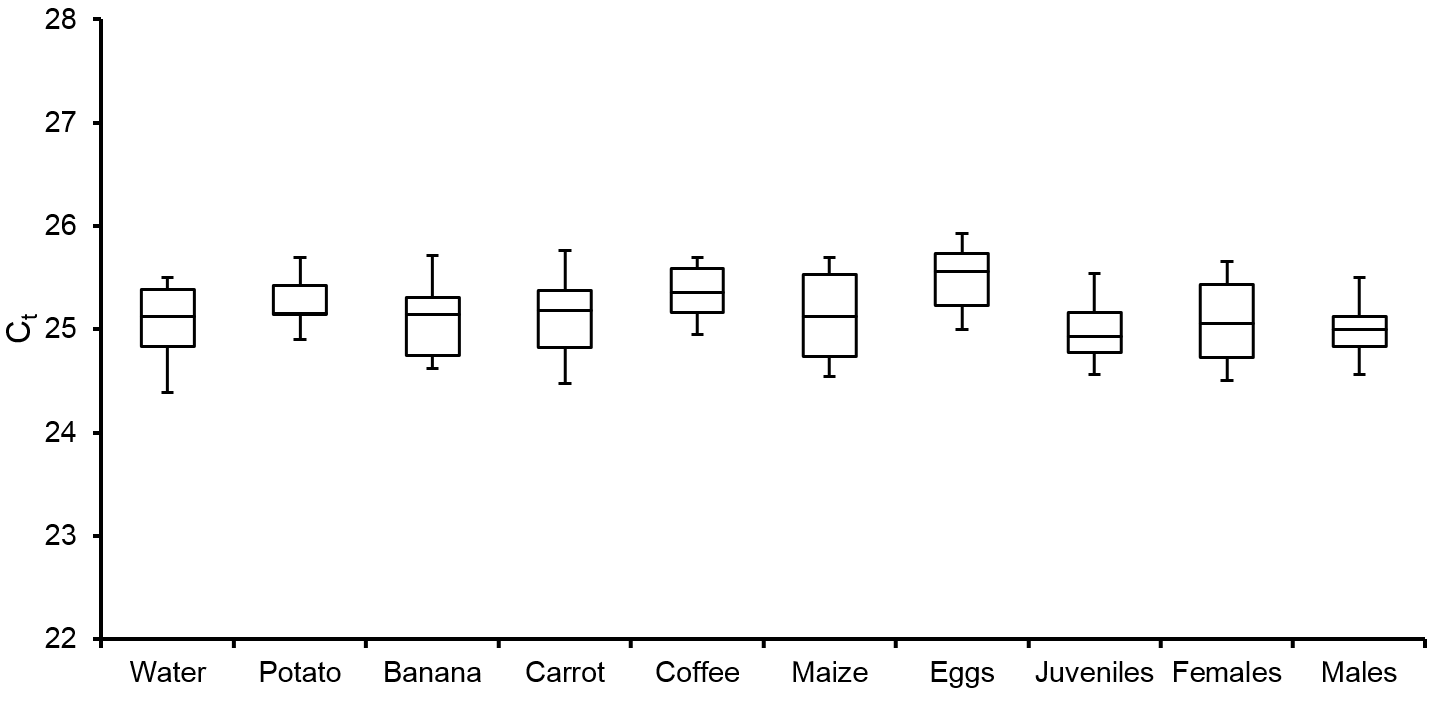

Supplement: S3 Fig — Values are means ± SEM (n = 4 pools of mixed stages). (DOCX) [file ppat.1007503.s003.docx]
